# Cytotoxicity of *Aspergillus* Section *Fumigati* Isolated from Health Care Environments

**DOI:** 10.3390/jof7100839

**Published:** 2021-10-07

**Authors:** Carla Viegas, Magdalena Twarużek, Beatriz Almeida, Marta Dias, Edna Ribeiro, Elisabete Carolino, Ewelina Soszczyńska, Liliana Aranha Caetano

**Affiliations:** 1 H&TRC—Health & Technology Research Center, ESTeSL—Escola Superior de Tecnologia da Saúde, Instituto Politécnico de Lisboa, 1990-086 Lisbon, Portugal; beatrizltalmeida1@gmail.com (B.A.); martasfd@gmail.com (M.D.); edna.ribeiro@estesl.ipl.pt (E.R.); etcarolino@estesl.ipl.pt (E.C.); liliana.caetano@estesl.ipl.pt (L.A.C.); 2 Public Health Research Centre, NOVA National School of Public Health, Universidade NOVA de Lisboa, 1099-085 Lisbon, Portugal; 3 Comprehensive Health Research Center (CHRC), NOVA National School of Public Health, Universidade NOVA de Lisboa, 1099-085 Lisbon, Portugal; 4 Department of Physiology and Toxicology, Faculty of Biological Sciences, Kazimierz Wielki University, Chodkiewicza 30, 85-064 Bydgoszcz, Poland; twarmag@ukw.edu.pl (M.T.); eweso@ukw.edu.pl (E.S.); 5 Research Institute for Medicines (iMed.Ulisboa), Faculty of Pharmacy, University of Lisbon, 1649-004 Lisbon, Portugal

**Keywords:** *Aspergillus* section *Fumigati*, health care environment, cytotoxicity, lung epithelial cells

## Abstract

This study analyzed 57 *Aspergillus* section *Fumigati* (AF) isolates collected by active and passive sampling (N = 450) in several health care facilities and from biological sampling of health care workers (N = 25) and controls (N = 22) in Portugal. All isolates were cultured in different media and screened for azole resistance. Cytotoxicity was assessed for 40 isolates in lung epithelial cells and kidney cells using the MTT assay. *Aspergillus* section *Fumigati* was prevalent in the health care facilities and in nasal swabs from health care workers and controls. All AF isolates reduced cell viability and presented medium to high cytotoxicity, with cytotoxicity being significantly higher in A549 lung epithelial cells. The cytotoxicity of isolates from air and nasal swab samples suggested the inhalation route as a risk factor. Notably, 42% of AF isolates exhibited a pattern of reduced susceptibility to some of the most used antifungals available for the treatment of patients infected with these fungi. In sum, the epidemiology and clinical relevance of *Aspergillus* section *Fumigati* should continue to be addressed. A deeper understanding of the mechanisms underlying *Aspergillus*-mediated cytotoxicity is necessary.

## 1. Introduction

*Aspergillus* section *Fumigati* is associated with a high mortality rate in at risk patients, such as those with asthma, cystic fibrosis, and chronic obstructive lung disease or with immune suppression, mostly due to invasive pulmonary aspergillosis—a fatal disease [1]. It is predictable that over 30 million patients are at risk of developing invasive aspergillosis worldwide, mainly due to the use of immunosuppressive therapies [2]. Healthcare-associated aspergillosis is most often acquired by inhalation of airborne spores causing pulmonary aspergillosis, which the fungus can disseminate in the bloodstream and reach other organs [3]. Airway colonization by *Aspergillus* spp. Has also been observed in approximately half of patients in an adult pneumology ward with no symptoms of aspergillosis [4]. Exposure to *Aspergillus* section *Fumigati* can be, therefore, considered a risk for both patients and health staff in health care environments (HCE) [5]. 

The genus *Aspergillus* is classified into four subgenera (*Aspergillus*, *Circumdati*, *Fumigati*, and *Nidulantes*) and 20 sections, each including a number of related species [6,7]. The most prevalent in HCE, as in other environments, are *Aspergillus* sections *Fumigati*, *Flavi*, *Nigri*, and *Nidulantes* [8,9]. Section *Fumigati* is the most frequently isolated section both from respiratory samples from patients and from HCE sampling [10]. Section *Fumigati* is one of the most species-rich sections in the *Aspergillus* genus, comprising about 50 to 60 potentially pathogenic species for humans [11]. It is the *Aspergillus* section that has the most reported clinical relevance and is more often associated with respiratory symptoms (mainly *A. fumigatus* sensu stricto). 

The adverse effects resulting from exposure to airborne toxigenic fungi are well known. Long-term exposure to toxigenic fungi interferes with natural killer cell activity, and may cause symptoms such as cough, fever, headache, anxiety, or depression [12]. The induction of immunosuppression and inflammation by exposure to fungi and bioaerosols is also described [13,14]. An additional concern regarding *A. fumigatus* sensu stricto is the emergence of acquired azole-resistance in clinical practice and in the environment [10,15,16,17,18,19]. 

*Aspergillus* section *Fumigati*’s clinical relevance has been related to the small size of conidia and other virulence factors [9,20,21]. The cytotoxicity effect of *Aspergillus* section *Fumigati* toxins, such as gliotoxin, has been described [22,23,24]. While a more recent study described cytotoxic and apoptotic effects of *Aspergillus* section *Fumigati* conidia in lung epithelial cells and fibroblasts [25]. 

In the present study, to evaluate the cytotoxicity of *Aspergillus* section *Fumigati* from ten primary health care centers and one central hospital, isolates were obtained by air, passive sampling and workers’ nasal swabbing and were co-cultured with lung epithelial cells and kidney cells. An MTT assay was used to determine IC50 levels, and correlational statistical analysis was performed to explore relations between isolates growth in different media, including azoles, sampling, and the cytotoxicity effect.

## 2. Materials and Methods

### 2.1. Health Care Facilities and Sampling Campaign

Ten Primary Health Care Centres (PHCC) and one Central Hospital (CH) were assessed in Lisbon and Oporto, respectively, from June to July 2018, as part of a wider study aiming to propose new procedures to determine exposure to bioburden at HCE [26]. The project protocol was first approved by scientific councils from HCE (ref: 064/CES/INV/2017) and by the Ethical Committee of Escola Superior de Tecnologia da Saúde de Lisboa (ref: CE-ESTeSL-No 45-2018). The protocol was in accordance with the World Medical Association Declaration of Helsinki and the Oviedo Convention, and in agreement with the Portuguese law no 58/2019 of 8 August, regarding data protection [26]. A prior evaluation by a certified exposure assessor was developed on site at each HCE to identify critical control points in workplaces which could involve higher exposure to microbial contamination. A comprehensive sampling campaign was then held, using active and passive sampling methods in both indoor environments (PHCC and CH) [27,28]. Active sampling comprised air sampling by impaction (N = 201). Air sampling by impinger was also performed (N = 56) for molecular detection purposes (not presented). Passive sampling included surface swabs (N = 126), electrostatic dust cloths (EDC, N = 96), settled dust (N = 15), and filters from HVAC system (N = 12) (Table 1).

### 2.2. Volunteers Enrolment and Biological Sampling

Nasal swabs were collected from volunteer health care workers in the ten PHCC (N = 25) and in the CH (N = 22). A control group of 25 healthy volunteers with no occupational contact with health care facilities was also evaluated in Lisbon. All volunteers signed informed consent prior to enrollment in the study. All inherent ethical principles were duly observed. Biological samples were obtained through a nasopharyngeal swab procedure using transport swabs with Stuart media when necessary. For nasal sampling, a swab was inserted about one centimeter into the nostril and rubbed in a circular way. The same swab was then used to sample the other nostril following the same procedure. For the samples collection, transport swabs with Stuart media were used that were immediately transported to the laboratory after being used.

### 2.3. Fungal Culture and Screening of Azole Resistance

Malt extract agar (MEA) supplemented with chloramphenicol (0.05%), and dichloran-glycerol agar (DG18) were used to increase selectivity for fungal growth. Sabouraud dextrose agar (SDA) and SDA supplemented with 4 mg/L itraconazole (ITR), 1 mg/L voriconazole (VOR) or 0.5 mg/L posaconazole (POS) were used for screening of azole resistance (adapted from EUCAST 2018) and following the procedures already reported [5,18,19]. The reference strain *A. fumigatus* ATCC 204,305 was used as a negative control and a pan-azole-resistant strain was used as a positive control (both kindly provided by Reference Unit for Parasitic and Fungal Infections, Department of Infectious Diseases of the National Institute of Health, from Dr. Ricardo Jorge).

After incubation at 27 °C for 5 to 7 days (MEA and DG18) and 27 °C for 4 days (SDA, ITR, VOR, and POS), fungal burden densities found in environmental samples (colony-forming units, CFU/m^2^) were calculated as previously described [18,27,28]. Fungal species were identified microscopically using tease mount or Scotch tape mount and lactophenol cotton blue mount procedures. Morphological identification was achieved through macro and microscopic characteristics as noted by De Hoog [29].

### 2.4. Aspergillus Section Fumigati Isolation

After identifying the *Fumigati* section in any of the media used, an isolate was obtained from each sample in MEA, and the one with the highest possibility of obtaining a pure culture of the *Fumigati* isolate was selected. *Aspergillus* section *Fumigati* isolates in MEA were retested in azole supplemented media. Before the cytotoxicity assay, *Aspergillus* section *Fumigati* was inoculated on the yeast extract glucose chloramphenicol (YGC) medium in order to revive the colony. Of note, some *Fumigati* isolates were unable to grow at this stage and could not be further analyzed. Then the isolates were inoculated on the Czapek’s agar (CZA) medium (final pH = 6.0 ± 0.2, at 25 °C) and grown for 10 days at 25 °C and 10 days at 6 °C. The composition of the CZA medium was as follows: sucrose-30.00 g/L, sodium nitrate-3.00 g/L, dipotassium phosphate-1.00 g/L, potassium chloride-0.50 g/L, magnesium sulphate-0.50 g/L, ferrous sulphate-0.01 g/l, Agar-15.00 g/L.

### 2.5. Cell Culture

Human A549 lung epithelial cells and swine kidney (SK) cells were maintained in Eagle’s minimum essential medium (MEM) supplemented with 10,000 units of penicillin and 10 mg of streptomycin per mL in 0.9% NaCl (Sigma-Aldrich, Portugal)) and fetal bovine serum (Sigma-Aldrich, USA). Cells were detached from the bottom of the culture vessel with 0.25% (*w*/*v*) Trypsin 0.53 mM EDTA, suspended in the culture medium, and the number of cells was counted using Scepter™ 2.0 cell counter (Merck).

### 2.6. Cytotoxicity Evaluation by the MTT Assay

The cytotoxicity effect was measured by reduction of MTT tetrazolium salt to formazan at 510 nm (Hanelt et al. 1994) in A549 and SK cells, using several dilutions of *Aspergillus* section *Fumigati* isolates. *Fumigati* isolates were exposed to thermal shock by being placed in the temperature of 4 °C for 96 h. From the strains of molds grown in the Petri dishes (Czapek-Dox medium) extracts were prepared to be evaporated later to dryness under a stream of nitrogen. The extracts contained the equivalent of *Aspergillus* section *Fumigati* from one Petri dish (62.5 cm^2^). Next, a series of test dilutions was prepared. The first dilution on assay plate was 31.25 cm^2^/mL. After the cell count, A549 and SK cells were transferred (100 µL) to a 96-well plate (densities of 2.5 × 10^5^ cells/mL) and exposed to the several dilutions of *Aspergillus* section *Fumigati* isolates for 48 h at 5% CO_2_, 37 °C, and humid atmosphere. The lowest concentration of the isolates causing a drop in absorption to <50% of cell division activity (IC50) was considered the threshold toxicity level.

### 2.7. Statistical Analysis

Data were analyzed using SPSS V26.0 statistical software for windows. The results were considered significant at the 5% significance level. To characterize the sample, frequency analysis (n, %) was used for qualitative data and graphical representations appropriate to the nature of the data. To test the normality of the data, the Shapiro-Wilk test was used. To study the association between the growth of azoles (no/yes in ITR, VOR, and POS) and the medium (MEA, DG18, and SAB) the Chi-Square test by Monte Carlo simulation was used, since the assumptions of applicability of the Chi-Square test were not verified. To compare the cytotoxicity of IC50 cells (SK and A549) between the MEA and DG18 media (once in the SDA media there were only three observations) and between the growth of azoles (no/yes in ITR, VOR, and POS) the Mann–Whitney test was used. To compare the cytotoxicity of IC50 cells (SK and A459) between the type of environmental samples (air impaction, nasal swabs (PHCC), and nasal swabs (Control)—the others were not considered in the analysis, due to the small number of observations), a Kruskal-Wallis test was used.

## 3. Results

### 3.1. Aspergillus Section Fumigati Isolates

A total of 57 *Aspergillus* section *Fumigati* isolates were recovered from 450 environmental active and passive samples and 47 samples obtained by nasal swabbing. *Aspergillus* section *Fumigati* isolates were recovered with higher prevalence from DG18 (56.1%) and the samples obtained by air impaction, were the environmental samples where it was observed more frequently (8.1%) (Table 2).

**Figure 1:**
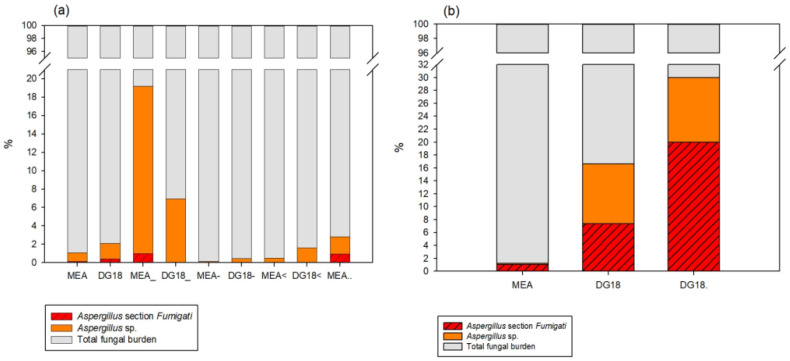
*Aspergillus* sp. and *Aspergillus* section *Fumigati* distribution in the PHCC samples (**a**) and CH samples (**b**) (Adapted from [5,27,28]).

*Aspergillus* section *Fumigati* was identified in all but one type of environmental sample (HVAC filters) in the PHCC studied. In the air impaction samples, this section was identified in both MEA and DG18, where it represented 0.13% and 0.41% of the total fungal burden, respectively. In all other types of environmental samples, *Aspergillus* section *Fumigati* was only detected in MEA, representing 0.01% of the total fungal burden in the EDCs, 1% in the surface swabs, and 0.94% in the settled dust samples. In the Central Hospital (CH), *Aspergillus* sp. was only identified in air impaction and settled dust samples. In the air impaction samples, *Aspergillus* section *Fumigati* was the most common section both in MEA (1.05%) and in DG18 (7.35%). The same trend was found on settled dust, where *Aspergillus* section *Fumigati* was the most common (20% of the total) (Figure 1).

### 3.2. Aspergillus Section Fumigati Cytotoxicity Effect

The cytotoxicity evaluation was obtained from 40 out of the 57 *Aspergillus* section *Fumigati* isolates, using the MTT assay. The lowest concentration of the isolates causing a drop in absorption to <50% of cell division activity was considered the threshold toxicity level. The overview of the results is shown in Table 3. The IC50 ranged from 0.7625 mm^2^/mL to 0.122 cm^2^/mL in A549 cells, and from 3.050 mm^2^/mL to 3.906 cm^2^/mL in SK cells.

A semi-quantitative scale for cytotoxicity grading was used (adapted from [30]): medium cytotoxic effect for IC50 values ranging from 3.906 cm^2^/mL to 0.977 cm^2^/mL; high cytotoxic effect for IC50 values ranging from 0.488 cm^2^/mL to 0.7625 mm^2^/mL. The results are depicted in Table 4. Cytotoxicity was confirmed in all *Aspergillus* section *Fumigati* isolates, with high cytotoxicity observed in 100% of cases in A549 lung epithelial cells, and in 95% of cases in SK cells. Similar results were obtained with isolates from nasal swabbing of workers and controls.

Of note, 17 *Aspergillus* section *Fumigati* isolates (88.2% from environmental sampling) were able to grow in at least one azole (4 mg/L ITR), including 7 isolates from environmental samples (mostly air impaction) that were able to grow in two different azoles (4 mg/L ITR, and 1 mg/L VOR), of which 4 were able to grow in the three tested azoles (4 mg/L ITR, 1 mg/L VOR, and 0.5 mg/L POS) [5,19,27,28]. Regarding nasal samples, two *Aspergillus* section *Fumigati* isolates were able to grow in 4 mg/L ITR, one of which was from PHCC staff, and the other from controls.

### 3.3. Correlation and Comparison Analysis

No significant correlation of IC50 levels was detected between SK and A549 cells (rS = 0.212, *p* = 0.209). Statistically significant differences in IC50 levels were detected between SK and A549 cells (z = −4.982, *p* < 0.001), with IC50 being significantly lower in A549 cells. The Wilcoxon test also revealed statistically significant differences of IC50 levels among the two cell types in different media (z = −3.413, *p* = 0.001 and z = −3.834, *p* < 0.001, in MEA and in DG18, respectively), with IC50 levels being significantly lower for both media in A549 cells (Table 5). Between MEA and DG18, no statistically significant differences in IC50 levels were detected in SK or A549 cells (U = 158, *p* = 0.705, and U = 156, *p* = 0.658, respectively) (Table 6). These results suggest a higher cytotoxicity effect in A549 cells, with no influence of the culture media used.

The Chi-Square test detected one significant association (*p* = 0.035, 95% C.I. = (0.031, 0.039)) between nongrowth in ITR and growth in MEA and DG18, with *Aspergillus* section *Fumigati* presenting a greater predisposition to nongrowth in ITR (Table 7). These results indicate that the three media analyzed (MEA, DG18, and SDA) present a significant variability with respect to fungal growth in ITR.

Comparing the cytotoxicity effect (IC50 levels in SK and A459 cells) with *Aspergillus* section *Fumigati* growth (No/Yes) on azole-supplemented media, no statistically significant differences were detected in any of the media (*p* > 0.05) (Table 8). These results suggest that cytotoxicity had no relation with *Aspergillus* section *Fumigati*’s ability to grow on azole-supplemented media. Despite not being significant, it was found that IC50 was lower (meaning higher cytotoxicity) in both cells when isolates were not able to grow in ITR or when they were able to grow in POS. Regarding VOR, IC50 was lower in SK cells for isolates grown in VOR, and in A549 cells when there was no growth in VOR (Table 8).

On the basis of the comparison of IC50 levels for individual isolates of the fungi, there were no statistically significant differences between IC50 levels of the analyzed samples (air impaction, nasal swab (PHCC) and nasal swab (control) for either SK ($\chi_{K-W}^{2}\left(2\right)$= 1.454, *p* = 0.483) or A459 ($\chi_{K-W}^{2}\left(2\right)=0$.514, *p* = 0.773)) cells. Therefore, it was not possible to clearly determine which of the compared sample type displays had a more cytotoxicity effect than others.

## 4. Discussion

In the present study, we exposed human lung epithelial A549 cells and swine kidney (SK) cells to *Aspergillus* section *Fumigati* isolates from the HCE and health care workers and found that all *Aspergillus* section *Fumigati* isolates tested reduced cell viability, presenting a medium to high cytotoxicity effect in culture. Human lung epithelial cells were used as a model for exposure by inhalation [31], and swine kidney cells as a model for mammal nephrotoxicity [32], considering the reported nephrotoxicity of some *Aspergillus* section *Fumigati* toxins [24].

Furthermore, we performed correlational statistical analysis and detected a higher cytotoxic effect in A549 cells, regardless the culture media used. This is particularly concerning regarding the cytotoxicity effect of *Aspergillus* section *Fumigati* isolates from air impaction samples and from nasal swabs of PHCC workers and controls, suggesting the inhalation route as a risk factor, especially for individuals suffering from asthma [33] and immunocompromised individuals [9,34,35]. 

Indeed, cytotoxic toxins of *Aspergillus* section *Fumigati* act on different cells to induce cell death. The cytotoxicity and apoptotic effects of gliotoxin, the main secondary metabolite of *Aspergillus* section *Fumigati* have been reported in macrophages [22,24]. Trypacidin, another toxin from *Aspergillus* section *Fumigati,* was also reported to have a cytotoxicity effect on lung cells [23]. Other studies using lung epithelial cells to address the association between *Fumigati* conidia and airways colonization revealed contradictory results on the pro-inflammatory effect [36,37].

Very few studies focus on the cytotoxicity of fungi from environments occupied by humans, namely, in HCE. Most available studies focus on the cytotoxicity of *Penicillium* sp., *Aspergillus* sp. or *Stachybotrys* sp. genera, mostly recovered from dwellings with infiltrations and humid environments [38,39,40,41,42], from occupational settings [43,44], or even from protection devices used in high fungal load settings such as the waste sorting industry [45,46,47]. One study evaluating the concentration of airborne fungi in rooms of asthma patients concluded that the home environment was a potential source of exposure to molds and a risk factor for asthma patients [33]. A previous study revealed that 47% of the evaluated airborne fungi, collected from humid apartments in Scotland, displayed cytotoxicity in vivo [38]. Other in vitro studies refer to the cytotoxicity of building materials as related to their contamination by molds and mycotoxins [48]. 

Regarding the cytotoxicity of the *Aspergillus* genera, a study by Gniadek et al. reported a low cytotoxicity effect of airborne *Aspergillus* section *Flavi* recovered from hospital rooms and tracheostomy tubes [49]. A previous study comparing the cytotoxicity of indoor molds, by means of the MTT assay, concluded that IC50 for *Aspergillus* section *Fumigati* spores was higher than for *Aspergillus* section *Nigri* spores [40], whereas several other studies describe that *Aspergillus* section *Fumigati* present the highest cytotoxicity among *Aspergillus* species [39,42], including one study in a hospital environment [50]. 

*Aspergillus* section *Fumigati* was more prevalent in DG18 (compared to MEA), thus, supporting the use of more than one culture media in HCE assessments. Indeed, DG18 restricts the size of fungal colonies with higher growth rates that often hinder *Aspergillus* sp. [51]. Therefore, a more accurate characterization of the contamination by this *Aspergillus* section should be considered [52]. 

The high prevalence of section *Fumigati* obtained by air impaction also highlights the risk of exposure by inhalation of airborne spores [3]. It is known that, when suspended in the air, the 2–3 μm conidia of this *Aspergillus* section can reach deeply inside the respiratory system (alveoli or the sinuses) after inhalation [53]. Previous work on health care units evaluated the air quality of indoor hospital environments, namely, in adult and new-born intensive care units, as well as surrounding areas such as corridors and hallways, concluding that fungal spores’ contamination was within limits (750 CFU.m^−3^) according to current norms [54]. Other studies, however, refer to fungal contamination in hospital room’s frequently exceeding limits [5,27,28,55,56,57]. In the enlarged project where the isolates from this study were recovered, the quantification limit complied with the Portuguese legislation in most of the HCE assessed (I/O < 1), which is the cut off to avoid fungal species identification. However, a deeper analysis enabled the identification of harmful fungal species (including section *Fumigati* among others), which are indicators for corrective measure implementation in the same Portuguese legal framework [27].

Besides fungal quantification suggested in most guidelines and legislation, the identification of toxigenic fungal species is also important for risk assessment [27,28]. Performing regular fungal assessments, targeting for *Aspergillus* section *Fumigati,* may help to unveil contamination sources at HCE [5,58]. Moreover, the sampling approach should comprise both active (air) and passive sampling methods and be adjusted to the identified contamination sources, contextual information, and variability of the exposure [52]. 

The quality guarantee of the HCE is aligned with the Sustainable Development Goals (SDGs), to ensure healthy lives and promote well-being for all at all ages (Goal 3) [59]. Studies held in European hospitals [60] reported that nosocomial infections significantly increase morbidity and mortality rates, with most of these infections being transmitted by airborne pathogens [61]. These specific indoor environments also present a high risk of cross infection between staff and patients. Additionally, fungal contamination in the air and on hospital surfaces has been associated with the number of fungal infections in hospitalized immunocompromised patients [9,34]. Monitoring and control of microbial contamination in HCE is, therefore, mandatory as it is crucial to prevent and control hospital-acquired infections [62,63]. Notably, several *Aspergillus* section *Fumigati* isolates from the environmental sampling were also able to grow in at least one azole, including isolates from air samples able to grow in two or more different azoles. This might be particularly critical in the HCE, where patients, visitors, and staff might be exposed, and in particular more susceptible populations, such as immunocompromised individuals [10,15,35]. Azole resistance must be confirmed in future studies, through antifungal susceptibility testing, for a more precise characterization of the relation between cytotoxicity and azole resistance of *Aspergillus* section *Fumigati* isolates collected in the environment of healthcare facilities. Unfortunately, the section *Fumigati* was classified based on macroscopic and microscopic characteristics and because of that it was impossible to identify the species among the section. However, in previous studies held by the same team also with environmental isolates from different indoor environments, results revealed a good correlation between phenotypic and molecular identification [19,64]. Further studies should comprehend molecular identification of *Aspergillus* isolates and cytotoxicity analyses.

## 5. Conclusions

In conclusion, *Aspergillus* section *Fumigati* was found to be a prevailing species in the assessed health care facilities and in nasal swab samples from health care workers. The presence of cytotoxic and azole resistant *Aspergillus* section *Fumigati* isolates in the HCE environment poses an additional risk for patients and health care workers, especially for immunocompromised individuals. The epidemiology and clinical relevance of this species should continue to be addressed, as a reduced susceptibility to azoles—some of the most used antifungals available—may lead to therapeutic failure in the treatment of fungal infections such as invasive aspergillosis. More studies on this topic are necessary to link *Aspergillus* section *Fumigati* cytotoxicity with nosocomial aspergillosis.

## Figures and Tables

**Table 1:** Samples collected in the HCE analyzed by culture-based methods (Adopted from [27,28]).

	Air Impaction	Surface Swabs	EDC	Settled Dust	HVAC Filters
PHCC	81	81	81	10	12
CH	120	45	15	5	0
Total	201	126	96	15	12
			450		

**Table 2:** Isolates (n) of *Aspergillus* section *Fumigati* recovered per sample collected and media applied for first isolation.

Media		Sample Collected							Total
		Air Impaction	EDC	Surface Swabs	Settled Dust	Vacuum Bag	Nasal Swab (PHCC)	Nasal Swab (Control)	
MEA	N	6	1	1	1	1	1	6	17
	% of total	10.5%	1.8%	1.8%	1.8%	1.8%	1.8%	10.5%	38.6%
DG18	N	16	0	0	1	0	8	16	41
	% of total	28.1%	0.0%	0.0%	1.8%	0.0%	14.0%	28.1%	56.1%
SDA	N	0	1	0	1	1	0	0	3
	% of total	0.0%	1.8%	0.0%	1.8%	1.8%	0.0%	0.0%	5.3%

**Table 3:** Distribution of threshold toxicity level (IC50) of *Aspergillus* section *Fumigati* isolates.

Dilution Step	IC50	A549	SK
		N	N
13	0.7625 mm^2^/mL	4	0
12	1.525 mm^2^/mL	9	0
11	3.050 mm^2^/mL	10	6
10	0.061 cm^2^/mL	14	10
9	0.122 cm^2^/mL	3	14
8	0.244 cm^2^/mL	0	3
7	0.488 cm^2^/mL	0	5
6	0.977 cm^2^/mL	0	1
4	3.906 cm^2^/mL	0	1

N, number of *Aspergillus* section *Fumigati* isolates toxic for A549 or SK cells.

**Table 4:** Level of cytotoxicity of the *Aspergillus* section *Fumigati* isolates.

*Aspergillus* Section *Fumigati* Isolates per Sampling	Isolates with Level of Toxicity n (%)			
	Medium		High	
	A549	SK	A549	SK
Air impaction (N = 13)	0	0	13 (100)	13 (100)
EDC (N = 1)	0	0	1 (100)	1 (100)
Settled dust (N = 3)	0	1 (33.3)	3 (100)	2 (66.7)
Surface swabs (N = 1)	0	0	1 (100)	1 (100)
Vacuum bag (N = 2)	0	0	2 (100)	2 (100)
Nasal swab (control) (N = 11)	0	1 (9.1)	11 (100)	10 (90.9)
Nasal swab (PHCC) (N = 9)	0	0	9 (100)	9 (100)

**Table 5:** Comparison of IC50 levels between SK and A549 cells from isolates first isolated on MEA and DG18. Wilcoxon test results.

			Ranks			Test Statistics	
			N	Mean rank	Sum of ranks	z	*p*
Global	A549-SK	Negative ranks	35 ^a^	18.56	649.50	−4.982 ^d^	0.000 *
		Positive ranks	1 ^b^	16.50	16.50		
		Ties	4 ^c^				
		Total	40				
MEA media	A549-SK	Negative ranks	15 ^a^	8.00	120.00	−3.413 ^d^	0.001 *
		Positive ranks	0 ^b^	0.00	0.00		
		Ties	2 ^c^				
		Total	17				
DG18 media	A549-SK	Negative ranks	19 ^a^	10.00	190.00	−3.834 ^d^	0.000 *
		Positive ranks	0 ^b^	0.00	0.00		
		Ties	1 ^c^				
		Total	20				

^a^. A549 < SK. ^b^. A549 > SK. ^c^. A549 = SK. ^d^. Based on positive ranks. * Statistically significant differences at the 5% significance level.

**Table 6:** Comparison of IC50 levels in either SK or A549 cells from isolates first isolated on MEA and DG18. Mann–Whitney test results.

IC50	Media	Ranks			Test Statistics	
		n	Mean Rank	Sum of Ranks	Mann–Whitney U	*p*
SK	MEA	17	19.71	335.00	158.000	0.705
	DG18	20	18.40	368.00		
	Total	37				
A549	MEA	17	19.82	337.00	156.000	0.658
	DG18	20	18.30	366.00		
	Total	37				

**Table 7:** Relation between *Aspergillus* section *Fumigati* isolates on azole-supplemented SDA (ITR, VOR, and POS) and first isolation media (MEA, DG18, and SDA). Results of the Chi-Square test by Monte Carlo simulation.

			Growth in Azole Supplemented Media				Chi-Square Test by Monte Carlo Simulation		
			ITR			Total	*p*	95% Confidence Interval	
			No		Yes			Lower Bound	Upper Bound
Media	MEA	n	12		10	22	0.035 ^a,^*	0.031	0.039
		%	54.5%		45.5%	100.0%			
	DG18	n	23		9	32			
		%	71.9%		28.1%	100,0%			
	SDA	n	0		3	3			
		%	0.0%		100.0%	100.0%			
Total			n	35	22	57			
			%	61.4%	38.6%	100.0%			
VOR									
Media	MEA	n	17		5	22	0.119 ^a^	0.112	0.125
		%	77.3%		22.7%	100.0%			
	DG18	n	30		2	32			
		%	93.8%		6.3%	100.0%			
	SDA	n	2		1	3			
		%	66.7%		33.3%	100.0%			
Total			n	49	8	57			
			%	86.0%	14.0%	100.0%			
POS									
Media	MEA	n	19		3	22	0.725 ^a^	0.716	0.734
		%	86.4%		13.6%	100.0%			
	DG18	n	30		2	32			
		%	93.8%		6.3%	100.0%			
	SDA	n	3		0	3			
		%	100.0%		0.0%	100.0%			
Total			n	52	5	57			
			%	91.2%	8.8%	100.0%			

^a^ Based on 10,000 sampled tables with starting seed 2,000,000. * Significant association at the 5% significance level.

**Table 8:** Comparison of IC50 levels (SK and A459 cells) between growth on azoles (No/Yes) in ITR, VOR, and POS media. Mann–Whitney test results.

Supplemented Media	IC50	Growth in the Azoles	Ranks			Test Statistics	
			n	Mean Rank	Sum of Ranks	Mann–Whitney U	*p*
ITR	SK	No	23	18.15	417.50	141.500	0.127
		Yes	17	23.68	402.50		
		Total	40				
	A549	No	23	30.68	422.50	146.500	0.164
		Yes	17	38.32	397.50		
		Total	40				
VOR	SK	No	33	20.80	686.50	105.500	0.713
		Yes	7	19.07	133.50		
		Total	40				
	A549	No	33	19.98	659.50	98.500	0.530
		Yes	7	22.93	160.50		
		Total	40				
POS	SK	No	36	21.04	757.50	52.500	0.364
		Yes	4	15.63	62.50		
		Total	40				
	A549	No	36	21.10	759.50	50.500	0.315
		Yes	4	15.13	60.50		
		Total	40				
